# Class-acquired influenza is more severe than non-class-acquired influenza: a survey for multi-school teachers

**DOI:** 10.3389/fpubh.2025.1662398

**Published:** 2025-10-07

**Authors:** Ruoqi Jin, Heng Zhao, Ying Ma, Shunjun Wu, Wanting Meng, Jing Li, Zhigui Cai, Ling Yang, Chenyao Li, Liqiang Song

**Affiliations:** Department of Respiratory and Critical Care Medicine, Xijing Hospital of Air Force Medical University, Xi'an, China

**Keywords:** influenza, respiratory infections, school teachers, epidemiologic studies, school health services

## Abstract

**Background:**

During the influenza season, teachers have a higher infection rate of influenza than the general population. However, there has been no study on the clinical characteristics of this specific influenza in infection type—“class-acquired influenza,” which means teachers acquire influenza infection from exposure to a substantial quantity of influenza virus aerosols in enclosed classrooms by teaching activities in a short period of time.

**Methods:**

In this study, teachers who had suffered from influenza during the 2024–2025 Seasonal Influenza from 12 schools in Xi'an, a city in northern China, were retrospectively collected through a questionnaire. They were divided into a class-acquired influenza group and a non-class-acquired influenza group, and then compared the clinical features and effects between these two groups.

**Results:**

Class-acquired cases showed higher incidence of symptoms, such as fever, cough, malaise, abdominal pain and diarrhea, and showed greater severity of symptoms like dry throat, runny nose and sputum. The class-acquired influenza group had longer recovery time [7.0 (5.0, 14.0) vs. 6.5 (5.0, 10.0) days, *p* = 0.003], and required more medical visits (69.9% vs. 52.3%, *p* = 0.004), medication (74.7% vs. 62.4%, *p* = 0.035), and sick leave (47.9% vs. 28.4%, *p* < 0.001). Incubation period (onset) showed no difference (*p* = 0.245).

**Conclusion:**

Class-acquired influenza is a specific type in teachers. Compared to non-class-acquired influenza, it involves worse symptoms, longer duration, higher healthcare needs, and more absenteeism, likely related to the unique working conditions and environment. Therefore, during the influenza season, schools and teachers should focus on rational prevention, early diagnosis and medication.

## 1 Introduction

Influenza is an acute infectious disease whose typical clinical manifestations (e.g., sore throat, nasal congestion and runny nose), systemic symptoms (e.g., fever and malaise), and gastrointestinal symptoms (e.g., abdominal pain and diarrhea) (1). Its epidemiology shows cyclical, seasonal and regional. Influenza epidemics often occur in winter and spring due to the temperate zone in most of China (2). Influenza is a self-limiting disease, but it still causes a huge disease burden worldwide (3). In China, it shows about 34,000 people need to visit hospitals due to the influenza virus every year (4), with about 88,100 influenza-associated excess respiratory deaths (5) and per capita medical expenditure is RMB 768.0–999.9 (6).

Influenza viruses infect all age groups, but school-aged children (5–18 years) exhibit particularly higher incidence rates (30–50%) compared with adults (7). Primary and secondary school students make up the majority of this population. Due to the long school hours, group activities and densely populated space among students, the influenza virus can easily spread rapidly in the influenza season (8). Not only students, but a few studies show teachers have a higher incidence of influenza than the general adult population (9). But there is no study on the clinical characteristics of influenza.

Teachers' susceptibility to influenza stems from a combination of environmental and occupational factors. In China, each classroom in primary and secondary schools keeps an average of 40~50 students. When influenza is highly prevalent in the cold weather, reduced ventilation in the classroom to maintain indoor temperatures leads to high viral aerosol density (10). Meanwhile, teachers spend about 90–130 h a week teaching. They even have to increase teaching duration for their sick colleagues in the influenza season (11). Additionally, teachers' occupational characteristics (elevated respiratory rate, high tidal volume, continuously open upper respiratory tract and lack of respiratory protection) significantly increase the risk of influenza infection when exposed to students who have influenza-like illness (ILI) in the classroom and expand the spread of influenza at school through teaching different classes.

This study is the first to use the term “class-acquired influenza” to describe teachers contracting influenza through teaching students with influenza. Compared with non-class-acquired influenza patients, class-acquired influenza cases may demonstrate shorter incubation periods, more severe symptoms and longer duration of illness, related to higher levels of influenza viruses inhaled in a shorter period of time through confined space, extended exposure time and high ventilation. However, there is no comprehensive study on this particular type of influenza.

This study investigated class-acquired influenza among primary and secondary school teachers in Xi'an, a representative city in northern China, during the 2024–2025 influenza season. Using questionnaire data from laboratory-confirmed influenza teachers, this study retrospectively collected and analyzed the characteristics and adverse effects of class-acquired influenza and conducted comparative analyses with non-class-acquired influenza teachers. The goal of this study is to provide a reference basis for the prevention and control program for this influenza infection among the teacher population.

## 2 Methods

### 2.1 Study design and setting

According to data from the Chinese Center for Disease Control and Prevention (CDC), the 2024–2025 influenza season in northern China spanned October 1, 2024 to April 13, 2025 (12). This retrospective study recruited teachers from 12 schools, one primary and one secondary school within each of the six main urban areas in Xi'an, Shaanxi Province. All laboratory-confirmed influenza cases among these teachers were invited to complete electronic questionnaires by sending a notification to their schools.

To ensure an accurate sample size estimate for this multi-center retrospective study, a pilot survey was initially conducted at one participating school. The pilot site had 98 teachers, of whom 17 met all inclusion criteria. The pilot data showed an influenza incidence rate of 17.3% during the observation period. Using PASS software, a minimum sample size of 238 participants was calculated for the main study, based on a margin of error of 5% and a statistical power (1 – β) of 0.80.

### 2.2 Quality assessment

To ensure data quality and minimize selection bias, we formed a survey team consisting of clinicians, school principals and administrators to guarantee all laboratory-confirmed influenza teachers received and completed the questionnaire.

### 2.3 Participants

All participating teachers provided informed consent to this survey and had laboratory-confirmed influenza infection during the study period. According to the Clinical Practice Guidelines for Influenza published by WHO (13), influenza is confirmed by a positive throat swab pathogen test with ILI, including: (1) a positive influenza virus nucleic acid test. (2) Positive rapid antigen test for influenza virus with epidemiologic history for comprehensive judgment.

“Classroom-acquired influenza” was defined as laboratory-confirmed influenza if both of the following conditions were met: (1) the symptoms of influenza-like illness occurred within one maximal incubation period after the participant had taught a student subsequently confirmed to have influenza; and (2) during the incubation window, the participant reported no close contact with any other known influenza case outside the classroom. Consistent with previous epidemiological studies, the incubation period for influenza is 1–4 days (14). The upper limit of this range (4 days) was used for exposure stratification. And we divided all participating teachers into two groups: the class-acquired influenza group and the non-class-acquired influenza group.

One patient may have multiple infections in one influenza season. Prior infection can impact the clinical characteristics of subsequent infection. Based on these, this study only analyzed clinical characteristics of the first influenza illness during the study period.

### 2.4 Study instruments

In this study, a questionnaire was used to collect clinical characteristics and the impact of influenza infection on teachers in the 2024–2025 influenza season. The questionnaire included: (1) demographic characteristics: gender, age and BMI; (2) medical history: smoking history, underlying diseases and influenza vaccination history; (3) infectious source in the defined incubation window (4 days in this study), such as students in the classroom, family members, and community contacts, and activities in the incubation (teaching or other social activities); (4) clinical characteristics of the first influenza infection during the study period: incidence and severity of typical symptoms (fever, dry throat, sore throat, nasal congestion, runny nose, cough, coughing up sputum, wheezing, dizziness, headache, malaise, muscle aches and pains, abdominal pains, diarrhea, etc.) (15) and duration of the illness, etc. Severity of symptoms was self-assessed and recorded on a 4-point scale (0: none, 1: mild, 2: moderate, 3: severe); (5) impact: hospital visits, work efficiency decline and absence.

### 2.5 Statistical analysis

PASS 15 was used for sample size estimation. Continuous variables were tested for normality by the Shapiro–Wilk test. Continuous variables failing to fit a normal distribution were described by the median (interquartile range, IQR) and compared using the Mann–Whitney *U* test. Categorical variables were described by frequency (percentage) and compared by the chi-square test or Fisher's exact test. Statistical analysis was performed with SPSS 27.0. Statistical significance was defined as a two-sided *P* < 0.05.

## 3 Results

The 12 schools surveyed had 52~104 (73 ± 20) teachers each, with a total of 855 teachers. This retrospective study covered the influenza season from October 1, 2024, to April 13, 2025. Based on medical records from school clinics, the proportion of teachers presenting with influenza-like illness (ILI) ranged from 12~ 43% (mean 35%), corresponding to about 300 cases of ILI patients. Two hundred and seventy-four laboratory-confirmed influenza cases were included in this study, accounting for 32.0% of all teachers and 91% of those with influenza-like illness. Two hundred and fifty-five questionnaires were reviewed and qualified, representing 29.8% of participants and 85% of symptomatic cases. Among the excluded questionnaires, 12 were invalid due to incomplete or unclear information (e.g., unknown source of infection) and 7 were excluded due to cross-infection with other pathogens.

Among them, 169 (66.3%) had 1 influenza infection during this influenza season, 75 (29.4%) had 2 infections during the period, and 11 (4.3%) had 3 or more infections. According to the grouping criteria, 146 patients were in the class-acquired influenza group and 109 cases were in the non-class-acquired influenza group. All of them were non-severe influenza.

### 3.1 Baseline characteristics

Table 1 demonstrated the baseline characteristics of patients in these two groups with class-acquired influenza and non-class-acquired influenza. Both groups were predominantly female, with more than 85% of the patients being female. The median age of patients in the class-acquired influenza group was 32.0 (27.0, 41.0) years, and the median age of patients in the non-class-acquired influenza group was 40 (31.0, 48.5) years, with the former group being slightly younger compared to the latter group (*P* < 0.001). There were no significant differences between the two groups in terms of BMI, smoking history, allergy history, history of underlying respiratory disease, or influenza vaccination rate (30.1% vs. 28.4%, *P* = 0.769). Of all the influenza cases collected, 142 (55.7%) were primary school teachers and 113 (44.3%) were secondary school teachers. There was a higher percentage of primary school teachers in the class-acquired influenza group compared with that of secondary school teachers (*P* = 0.009).

**Table 1:** Baseline characteristics of patients included in the study.

**Characteristics**	**Class-acquired (*n* = 146)**	**Non-class-acquired (*n* = 109)**	**Total (*n* = 255)**	***P*-value**
Gender, *n* (%)				0.212
Male	14 (9.6)	16 (14.7)	30 (11.8)	
Female	132 (90.4)	93 (85.3)	225 (88.2)	
Age, M (IQR)	32.0 (27.0, 41.0)	40.0 (31.0, 48.5)	36.0 (28.0, 45.0)	< 0.001
BMI, M (IQR)	22.0 (20.2, 24.8)	22.3 (20.2, 24.4)	22.0 (20.2, 24.6)	0.388
Smoking history, *n* (%)	4 (2.7)	8 (7.3)	12 (4.7)	0.086
Sleep apnea, *n* (%)	45 (31.0)	29 (26.6)	74 (29.1)	0.442
Allergy, *n* (%)	49 (33.8)	41 (37.6)	90 (35.4)	0.529
Pharyngeal discomfort, *n* (%)	66 (45.2)	50 (45.9)	116 (45.5)	0.916
Chronic cough or wheezing, *n* (%)	35 (24.0)	20 (18.3)	55 (21.6)	0.280
Received influenza vaccination, *n* (%)	44 (30.1)	31 (28.4)	75 (29.4)	0.769
School level				0.009
Elementary school	75 (51.4)	38 (34.9)	113 (44.3)	
Secondary school	71 (48.6)	71 (65.1)	142 (55.7)	

### 3.2 Symptom incidence

The findings to the clinical incidence of influenza symptoms between these two groups revealed that the incidence of fever, cough, malaise, and abdominal pain and diarrhea of patients in the class-acquired influenza group was higher than that in the non-class-acquired influenza group, including the incidence of fever being 67.8% and 45.0% (*P* < 0.001), the incidence of cough being 93.2% and 83.5% (*P* = 0.015), the incidence of malaise being 93.2% and 80.7% (*P* = 0.003), and the incidence of abdominal pain and diarrhea was 52.7% and 33.0% (*P* = 0.002). The incidence of the other symptoms was not significantly different between the two groups (Table 2).

**Table 2:** Incidence of each symptom in both groups of patients.

**Symptoms**	**Class-acquired (*n* = 146)**	**Non-class-acquired (*n* =1 09)**	**Total (*n* = 255)**	***P*-value**
Fever, *n* (%)	99 (67.8)	49 (45.0)	148 (58.0)	< 0.001
Sore throat, *n* (%)	128 (87.7)	97 (89.0)	225 (88.2)	0.746
Dry throat, *n* (%)	127 (87.0)	85 (78.0)	212 (83.1)	0.057
Nasal congestion, *n* (%)	121 (82.9)	84 (77.1)	205 (80.4)	0.247
Runny nose, *n* (%)	123 (84.2)	83 (76.1)	206 (80.8)	0.104
Cough, *n* (%)	136 (93.2)	91 (83.5)	227 (89.0)	0.015
Sputum, *n* (%)	127 (87.0)	85 (78.0)	212 (83.1)	0.057
Wheezing, *n* (%)	89 (61.0)	57 (52.3)	146 (57.3)	0.166
Dizziness, *n* (%)	102 (69.9)	69 (63.3)	171 (67.1)	0.270
Headache, *n* (%)	111 (76.0)	79 (72.5)	190 (74.5)	0.520
Fatigue, *n* (%)	136 (93.2)	88 (80.7)	224 (87.8)	0.003
Muscle aches, *n* (%)	123 (84.2)	84 (77.1)	207 (81.2)	0.147
Abdominal pain or diarrhea, *n* (%)	77 (52.7)	36 (33.0)	113 (44.3)	0.002

### 3.3 Severity of symptoms

In the quantitative or semi-quantitative comparison with the severe level of clinical symptoms between the two groups, patients in the class-acquired influenza group had a more severe degree of fever, dry throat, runny nose, sputum, abdominal pain and diarrhea compared to the non-class-acquired influenza group (*P* < 0.05) (Table 3).

**Table 3:** Severity of each symptom in both groups of patients.

**Symptoms**	**Class-acquired (*n* = 146)**	**Non-class-acquired (*n* = 109)**	**Total (*n* = 255)**	***P*-value**
Temperature, *n* (%)				< 0.001
37.3–38.0 °C	33 (22.6)	21 (19.3)	54 (21.2)	
38.1–39.0 °C	49 (33.6)	20 (18.3)	69 (27.1)	
>39.0 °C	17 (11.6)	8 (7.3)	25 (9.8)	
Sore throat, *n* (%)				0.315
Mild	36 (24.7)	38 (34.9)	74 (29.0)	
Moderate	49 (33.6)	31 (28.4)	80 (31.4)	
Severe	43 (29.5)	28 (25.7)	71 (27.8)	
Dry throat, *n* (%)				0.007
Mild	44 (30.1)	38 (34.9)	82 (32.2)	
Moderate	48 (32.9)	33 (30.3)	81 (31.8)	
Severe	35 (24.0)	14 (12.8)	49 (19.2)	
Nasal congestion, *n* (%)				0.075
Mild	31 (21.2)	30 (27.5)	61 (23.9)	
Moderate	44 (30.1)	27 (24.8)	71 (27.8)	
Severe	46 (31.5)	27 (24.8)	73 (28.6)	
Runny nose, *n* (%)				0.024
Mild	38 (26.0)	31 (28.4)	69 (27.1)	
Moderate	46 (31.5)	35 (32.1)	81 (31.8)	
Severe	39 (26.7)	17 (15.6)	56 (22.0)	
Cough, *n* (%)				0.060
Mild	40 (27.4)	28 (25.7)	68 (26.7)	
Moderate	41 (28.1)	31 (28.4)	72 (28.2)	
Severe	55 (37.7)	32 (29.4)	87 (34.1)	
Sputum, *n* (%)				0.011
Mild	46 (31.5)	38 (34.9)	84 (32.9)	
Moderate	48 (32.9)	34 (31.2)	82 (32.2)	
Severe	33 (22.6)	13 (11.9)	46 (18.0)	
Wheezing, *n* (%)				0.146
Mild	44 (30.1)	28 (25.7)	72 (28.2)	
Moderate	26 (17.8)	22 (20.2)	48 (18.8)	
Severe	19 (13.0)	7 (6.4)	26 (10.2)	
Dizziness, *n* (%)				0.216
Mild	46 (31.5)	35 (32.1)	81 (31.8)	
Moderate	38 (26.0)	22 (20.2)	60 (23.5)	
Severe	18 (12.3)	12 (11.0)	30 (11.8)	
Headache, *n* (%)				0.104
Mild	37 (25.3)	39 (35.8)	76 (29.8)	
Moderate	48 (32.9)	24 (22.0)	72 (28.2)	
Severe	26 (17.8)	16 (14.7)	42 (16.5)	
Fatigue, *n* (%)				0.077
Mild	46 (31.5)	28 (25.7)	74 (29.0)	
Moderate	50 (34.2)	36 (33.0)	86 (33.7)	
Severe	40 (27.4)	24 (22.0)	64 (25.1)	
Muscle aches				0.287
Mild	42 (28.8)	28 (25.7)	70 (27.5)	
Moderate	38 (26.0)	28 (25.7)	66 (25.9)	
Severe	43 (29.5)	28 (25.7)	71 (27.8)	
Abdominal pain or diarrhea, *n* (%)				0.002
Mild	45 (30.8)	23 (21.1)	68 (26.7)	
Moderate	26 (17.8)	10 (9.2)	36 (14.1)	
Severe	6 (4.1)	3 (2.8)	9 (3.5)	

### 3.4 Clinical outcomes

From the comparison of clinical outcomes between the two groups of cases, the findings revealed that the median influenza incubation period was 2.0 (1.0, 3.0) days in both groups, with no statistically significant difference (*P* = 0.245). The duration of illness was longer in the class-acquired influenza group than that in the non-influenza-acquired influenza group, with medians of 7.0 (5.0, 14.0) days vs. 6.5 (5.0, 10.0) days, with a statistically significant difference (*P* = 0.003). A higher percentage of the class-acquired influenza group required Medical institution treatment (69.9% vs. 52.3%, *P* = 0.004). Similarly, a higher proportion of this group required medication (74.7% vs. 62.4%, *P* = 0.035), especially antiviral drugs (39.0% vs. 26.6%, *P* = 0.038). Comparing the impact of influenza on work between the two groups, the impact was heavier in the class-acquired influenza group, as evidenced by a higher percentage of absence (47.9% vs. 28.4%) (Table 4).

**Table 4:** Clinical outcomes in both groups.

**Outcomes**	**Class-acquired (*n* = 146)**	**Non-class-acquired (*n* = 109)**	**Total (*n* = 255)**	***P*-value**
Incubation period, M (IQR)	2.0 (1.0, 3.0)	2.0 (1.0, 3.0)	2.0 (1.0, 3.0)	0.245
Disease recovery time, M (IQR)	7.0 (5.0, 14.0)	6.5 (5.0, 10.0)	7.0 (5.0, 10.0)	0.003
Medical institution treatment, *n* (%)	102 (69.9)	57 (52.3)	159 (62.4)	0.004
Medication	109 (74.7)	68 (62.4)	177 (69.4)	0.035
Antiviral drugs	57 (39.0)	29 (26.6)	86 (33.7)	0.038
Antibiotics	52 (35.6)	36 (33.0)	88 (34.5)	0.667
Symptomatic treatment	66 (45.2)	60 (55.0)	126 (49.4)	0.120
Impact on work, *n* (%)				< 0.001
No influence	5 (3.4)	22 (20.2)	27 (10.6)	
Less influence	71 (48.6)	56 (51.4)	127 (49.8)	
Absence	70 (47.9)	31 (28.4)	101 (39.6)	

M, median; IQR, inter-quartile range.

### 3.5 Clinical differences between elementary school and secondary school

**Figure 1:**
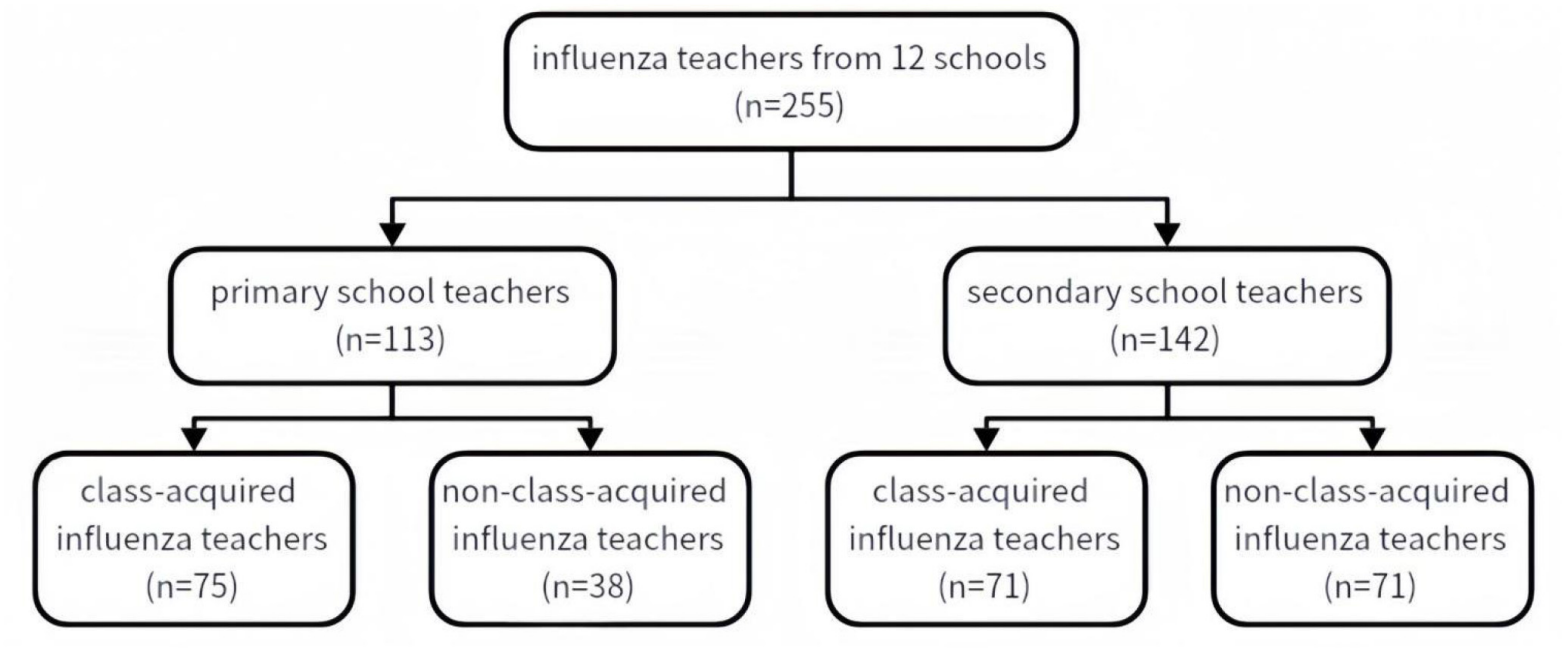
Subgroup analysis pipeline.

Figure 1 demonstrated further subgroup analyses between primary and secondary schools. In Supplementary Table S1 showed the comparison with the prevalence of influenza symptoms. Among primary school teachers, there was no significant difference between the two groups. In contrast, among secondary school teachers, the prevalence of fever, dry throat, malaise, abdominal pain and diarrhea was higher in patients in the class-acquired influenza group than that in the non-class-acquired influenza group, respectively 63.4% vs. 39.4%, *P* = 0.004; 88.7% vs. 73.2%, *P* = 0.019; 95.8% vs. 81.7%, *P* = 0.008; 47.9% vs. 26.8%. *P* = 0.009. The remaining symptoms were not significantly different between groups.

Supplementary Table S2 compared the symptom severity. Among primary school teachers, there was no significant difference between the two groups in the severity of individual symptoms. In secondary school, however, teachers in the class-acquired influenza group had more severe symptoms such as fever, dry throat, cough, sputum, abdominal pain and diarrhea than those in the non-class-acquired influenza group.

Supplementary Table S3 demonstrated the differences in disease outcomes. In terms of disease duration, there was no significant difference between the two groups of primary school teachers. Among secondary school teachers, patients in the class-acquired influenza group had a longer disease duration, 7.0 (5.0, 15.0) vs. 5.0 (5.0, 7.0), *P* < 0.001. Among primary school teachers, a higher proportion of cases in the class-acquired influenza group received medication (76.0% vs. 39.5%, *P* < 0.001), whereas there was no significant difference between the two groups of secondary school teachers. Among primary school teachers, there was no significant difference between the two groups of patients in the impact of the disease on their work. However, among secondary school teachers, cases in the class-acquired influenza group had a heavier impact on their work.

## 4 Discussion

Schools are high-risk areas for influenza virus transmission during the influenza season. Densely populated classrooms with relatively confined environments and poor ventilation make influenza virus-laden droplets and aerosols from coughing, sneezing, or talking suspend in the air, increasing the risk of transmission. It has been confirmed that the density of the airborne influenza virus in classrooms during the influenza season can reach the transmission threshold (16). Under these objective environmental conditions, both students and teachers are at high risk of influenza infection. Teachers, in particular, face elevated occupational exposure due to frequent speaking, open airways and inability to mask (17).

This study first introduced the concept of “class-acquired influenza” in teachers. “Classroom-acquired influenza” was the laboratory-confirmed influenza in teachers if the symptoms of influenza-like illness occurred within incubation period after the patients had taught a student subsequently confirmed to have influenza; and the teachers had no close contact with any other known influenza case outside the classroom during the incubation window. After comparative analyses of infected teachers in primary and secondary schools, we confirmed class-acquired influenza as a special influenza infection among the teacher population, revealing higher symptom severity and adverse effects than those of non-class-acquired influenza cases. These findings underscored that not only for students, society and schools should focus on protective measures, early diagnosis and treatment but also for teachers during the influenza season.

This study demonstrated that teachers with class-acquired influenza exhibited higher incidence and severity of respiratory symptoms compared to the non-class-acquired group. Specifically, the class-acquired influenza cases had a high prevalence of cough symptoms and symptoms such as dry throat, runny nose and sputum were significantly more severe. This might be related to several factors: firstly, a group in Ethiopia found that teachers had a high prevalence of chronic underlying respiratory diseases (18), and virus-infected patients with chronic inflammatory diseases were associated with elevated viral loads (19). Secondly, owing to teachers' occupational specificity (e.g., prolonged speaking), mucosal hydration was reduced in the pharynx, inducing submucosal vasodilation and hyperemia (20). Thereby, a dry mucosal barrier facilitated viral invasion into the upper respiratory tract and elevated viral loads post-infection. Thirdly, high virus density in the classroom during the influenza season led to high viral exposure (10), and teachers continuously inhaled the virus. Fourthly, teaching increased tidal volume, potentially increasing viral inhalation in the lungs and prolonging pulmonary injury (20). In conclusion, chronic respiratory diseases, heightened viral susceptibility and sustained high viral loads were likely to exacerbate respiratory symptom severity in class-acquired influenza teachers.

This study also revealed a higher incidence and severity of systemic symptoms. The results showed the class-acquired influenza teachers had a higher incidence of malaise and a more severe level of malaise and muscle aches than the non-class-acquired influenza cases. These might be associated with high viral load and severe systemic inflammatory response to influenza infection, even mild viral sepsis. A study for hospitalized patients with COVID-19 demonstrated strong correlations between peak viral load and inflammatory markers (IL-6 and procalcitonin) with minimal lymphocyte values (21). An influenza study for healthcare workers also found that the probability of visiting a doctor varied by position, possibly related to occupational exposure level and viral load effect on symptomatology (22). In addition, another study showed the severity of systemic symptoms was also higher in the physician population than in the general population (22). All these phenomena may be related to factors such as environmental viral exposure and occupational fatigue.

This study also observed a higher incidence and severity of gastrointestinal (GI) symptoms in class-acquired influenza cases. Previous research found that GI symptoms were associated with viremia triggered by high viral load (23), while direct viral colonization of the GI tract was also a reason (24). The influenza virus in patients was evidenced by fecal viral detection (23). Therefore, we hypothesized that the virus can be swallowed into the GI tract with saliva during teaching, potentially explaining symptoms like abdominal pain and diarrhea (26).

Based on the analysis, teachers with class-acquired influenza might have high incidence and severity of clinical symptoms, long duration of illness, increased hospital visits and medication, and extended absenteeism. These outcomes were likely to the high viral load and occupational exposure. These findings underscored the necessity of recognizing teachers as a distinct occupational risk group, highlighting the importance of respiratory health care in class as an emerging public health topic.

Subgroup analysis revealed that in secondary school teachers, severity of illness was significantly higher in the class-acquired influenza than in non-class-acquired influenza, manifested through more severe clinical symptoms, longer duration of illness, and so on. Whereas, the primary reason was likely related to the insufficient sample size collected in the primary school group. A potential explanation might lie in medication patterns. In terms of medication, primary school teachers with classroom-acquired influenza showed higher medication rates, while secondary school teachers exhibited the opposite trend. Therefore, it was likely that early pharmacologic intervention for primary school teachers alleviated the condition of the class-acquired influenza. Similarly, previous investigations on hospitalized adults with influenza demonstrated that early empirical antiviral therapy improved clinical outcomes (25). However, it couldn't be ruled out that the behavioral differences observed between the two teacher groups might also be attributable to inadequate sample size. Future studies with more participants are needed to confirm these results.

This study has several limitations. First, this study was a retrospective research and the data may be influenced by recall bias. Second, only laboratory-diagnosed patients were collected, so the study was not able to estimate the prevalence of class-acquired influenza and non-class-acquired influenza among teachers. Third, the sample size of this study was relatively small, as the subgroup analyses for primary and secondary schools were inconsistent with the overall findings. Finally, China is a vast country with different climates in winter and spring between the north and south. The information was collected from a northern Chinese city, lack of the generalizability to southern China. These constraints highlight the need for future prospective, multi-regional studies to validate our findings.

## Data Availability

The raw data supporting the conclusions of this article will be made available by the authors, without undue reservation.
