# Supplementary material for: Class-acquired influenza is more severe than non-class-acquired influenza: a survey for multi-school teachers
Source: Front Public Health. 2025 Oct 7;13:1662398. doi: 10.3389/fpubh.2025.1662398 (PMC12537437; doi:10.3389/fpubh.2025.1662398)
Supplement: Supplementary file 1 [file Supplementary_file_1.docx]

Table S1 Incidence of each symptom in both groups of patients in elementary and secondary schools

| Symptoms | Elementary school | | | Secondary school | | |
| --- | --- | --- | --- | --- | --- | --- |
|  | Class-acquired (n=75) | Non class-acquired (n=38) | P value | Class-acquired (n=71) | Non class-acquired (n=71) | P value |
| Fever, n (%) | 54 (72.0) | 21 (55.3) | 0.075 | 45 (63.4) | 28 (39.4) | 0.004 |
| Sore throat, n (%) | 66 (88.0) | 33 (86.8) | 1.000 | 62 (87.3) | 64 (90.1) | 0.596 |
| Dry throat, n (%) | 64 (85.3) | 33 (86.8) | 0.828 | 63 (88.7) | 52 (73.2) | 0.019 |
| Nasal congestion, n (%) | 61 (81.3) | 28 (73.7) | 0.348 | 60 (84.5) | 56 (78.9) | 0.385 |
| Runny nose, n (%) | 61 (81.3) | 27 (71.1) | 0.214 | 62 (87.3) | 56 (78.9) | 0.179 |
| Cough, n (%) | 70 (93.3) | 32 (84.2) | 0.226 | 66 (93.0) | 59 (83.1) | 0.070 |
| Sputum, n (%) | 65 (86.7) | 28 (73.7) | 0.088 | 62 (87.3) | 57 (80.3) | 0.255 |
| Wheezing, n (%) | 50 (66.7) | 19 (50.0) | 0.086 | 39 (54.9) | 38 (53.5) | 0.866 |
| Dizziness, n (%) | 52 (69.3) | 24 (63.2) | 0.509 | 50 (70.4) | 45 (63.4) | 0.373 |
| Headache, n (%) | 56 (74.7) | 26 (68.4) | 0.482 | 55 (77.5) | 53 (74.6) | 0.694 |
| Fatigue, n (%) | 68 (90.7) | 30 (78.9) | 0.083 | 68 (95.8) | 58 (81.7) | 0.008 |
| Muscle aches, n (%) | 63 (84.0) | 31 (81.6) | 0.745 | 60 (84.5) | 53 (74.6) | 0.145 |
| Abdominal pain or diarrhea, n (%) | 43 (57.3) | 17 (44.7) | 0.205 | 34 (47.9) | 19 (26.8) | 0.009 |

Table S2 Severity of each symptom in both groups of patients in elementary and secondary schools

| Symptoms | Elementary school | | | Secondary school | | |
| --- | --- | --- | --- | --- | --- | --- |
|  | Class-acquired (n=75) | Non class-acquired (n=38) | P value | Class-acquired (n=71) | Non class-acquired (n=71) | P value |
| Temperature, n (%) |  |  | 0.186 |  |  | 0.002 |
| ＜37.3℃ | 21 (28.0) | 17 (44.7) |  | 26 (36.6) | 43 (60.6) |  |
| 37.3-38.0℃ | 16 (21.3) | 7 (18.4) |  | 17 (23.9) | 14 (19.7) |  |
| 38.1-39.0℃ | 29 (38.7) | 8 (21.1) |  | 20 (28.2) | 12 (16.9) |  |
| ＞39.0℃ | 9 (12.0) | 6 (15.8) |  | 8 (11.3) | 2 (2.8) |  |
| Sore throat, n (%) |  |  | 0.204 |  |  | 0.583 |
| None | 9 (12.0) | 5 (13.2) |  | 9 (12.7) | 7 (9.9) |  |
| Mild | 20 (26.7) | 18 (47.4) |  | 16 (22.5) | 20 (28.2) |  |
| Moderate | 26 (34.7) | 5 (13.2) |  | 23 (32.4) | 26 (36.6) |  |
| Severe | 20 (26.7) | 10 (26.3) |  | 23 (32.4) | 18 (25.4) |  |
| Dry throat, n (%) |  |  | 0.553 |  |  | 0.008 |
| None | 11 (14.7) | 5 (13.2) |  | 8 (11.3) | 19 (26.8) |  |
| Mild | 18 (24.0) | 12 (31.6) |  | 26 (36.6) | 26 (36.6) |  |
| Moderate | 28 (37.3) | 14 (36.8) |  | 20 (28.2) | 19 (26.8) |  |
| Severe | 18 (24.0) | 7 (18.4) |  | 17 (23.9) | 7 (9.9) |  |
| Nasal congestion, n (%) |  |  | 0.170 |  |  | 0.243 |
| None | 14 (18.7) | 10 (26.3) |  | 11 (15.5) | 15 (21.1) |  |
| Mild | 13 (17.3) | 11 (28.9) |  | 18 (25.4) | 19 (26.8) |  |
| Moderate | 25 (33.3) | 7 (18.4) |  | 19 (26.8) | 20 (28.2) |  |
| Severe | 23 (30.7) | 10 (26.3) |  | 23 (32.4) | 17 (23.9) |  |
| Runny nose, n (%) |  |  | 0.116 |  |  | 0.122 |
| None | 14 (18.7) | 11 (28.9) |  | 9 (12.7) | 15 (21.1) |  |
| Mild | 15 (20.0) | 8 (21.1) |  | 23 (32.4) | 23 (32.4) |  |
| Moderate | 25 (33.3) | 13 (34.2) |  | 21 (29.6) | 22 (31.0) |  |
| Severe | 21 (28.0) | 6 (15.8) |  | 18 (25.4) | 11 (15.5) |  |
| Cough, n (%) |  |  | 0.844 |  |  | 0.030 |
| None | 5 (6.7) | 6 (15.8) |  | 5 (7.0) | 12 (16.9) |  |
| Mild | 21 (28.0) | 8 (21.1) |  | 19 (26.8) | 20 (28.2) |  |
| Moderate | 24 (32.0) | 10 (26.3) |  | 17 (23.9) | 21 (29.6) |  |
| Severe | 25 (33.3) | 14 (36.8) |  | 30 (42.3) | 18 (25.4) |  |
| Sputum, n (%) |  |  | 0.291 |  |  | 0.015 |
| None | 10 (13.3) | 10 (26.3) |  | 9 (12.7) | 14 (19.7) |  |
| Mild | 26 (34.7) | 9 (23.7) |  | 20 (28.2) | 29 (40.8) |  |
| Moderate | 21 (28.0) | 13 (34.2) |  | 27 (38.0) | 21 (29.6) |  |
| Severe | 18 (240.0) | 6 (15.8) |  | 15 (21.1) | 7 (9.9) |  |
| Wheezing, n (%) |  |  | 0.155 |  |  | 0.733 |
| None | 25 (33.3) | 19 (50) |  | 32 (45.1) | 33 (46.5) |  |
| Mild | 24 (32.0) | 7 (18.4) |  | 20 (28.2) | 21 (29.6) |  |
| Moderate | 13 (17.3) | 9 (23.7) |  | 13 (18.3) | 13 (18.3) |  |
| Severe | 13 (17.3) | 3 (7.9) |  | 6 (8.5) | 4 (5.6) |  |
| Dizziness, n (%) |  |  | 0.473 |  |  | 0.408 |
| None | 23 (30.7) | 14 (36.8) |  | 21 (29.6) | 26 (36.6) |  |
| Mild | 22 (29.3) | 13 (34.2) |  | 24 (33.8) | 22 (31.0) |  |
| Moderate | 17 (22.7) | 3 (7.9) |  | 21 (29.6) | 19 (26.8) |  |
| Severe | 13 (17.3) | 8 (21.1) |  | 5 (7.0) | 4 (5.6) |  |
| Headache, n (%) |  |  | 0.290 |  |  | 0.229 |
| None | 19 (25.3) | 12 (31.6) |  | 16 (22.5) | 18 (25.4) |  |
| Mild | 19 (25.3) | 13 (34.2) |  | 18 (25.4) | 26 (36.6) |  |
| Moderate | 20 (26.7) | 5 (13.2) |  | 28 (39.4) | 19 (26.8) |  |
| Severe | 17 (22.7) | 8 (21.1) |  | 9 (12.7) | 8 (11.3) |  |
| Fatigue, n (%) |  |  | 0.309 |  |  | 0.221 |
| None | 7 (9.3) | 8 (21.1) |  | 3 (4.2) | 13 (18.3) |  |
| Mild | 20 (26.7) | 9 (23.7) |  | 26 (36.6) | 19 (26.8) |  |
| Moderate | 24 (32.0) | 10 (26.3) |  | 26 (36.6) | 26 (36.6) |  |
| Severe | 24 (32.0) | 11 (28.9) |  | 16 (22.5) | 13 (18.3) |  |
| Muscle aches |  |  | 0.559 |  |  | 0.597 |
| None | 12 (16.0) | 7 (18.4) |  | 11 (15.5) | 18 (25.4) |  |
| Mild | 16 (21.3) | 10 (26.3) |  | 26 (36.6) | 18 (25.4) |  |
| Moderate | 21 (28.0) | 9 (23.7) |  | 17 (23.9) | 19 (26.8) |  |
| Severe | 26 (34.7) | 12 (31.6) |  | 17 (23.9) | 16 (22.5) |  |
| Abdominal pain or diarrhea, n (%) |  |  | 0.157 |  |  | 0.011 |
| None | 32 (42.7) | 21 (55.3) |  | 37 (52.1) | 52 (73.2) |  |
| Mild | 26 (34.7) | 12 (31.6) |  | 19 (26.8) | 11 (15.5) |  |
| Moderate | 14 (18.7) | 4 (10.5) |  | 12 (16.9) | 6 (8.5) |  |
| Severe | 3 (4.0) | 1 (2.6) |  | 3 (4.2) | 2 (2.8) |  |

Table S3 Clinical outcomes in both groups of patients in elementary and secondary schools

| Outcomes | Elementary school | | | Secondary school | | |
| --- | --- | --- | --- | --- | --- | --- |
|  | Class-acquired (n=75) | Non class-acquired (n=38) | P value | Class-acquired (n=71) | Non class-acquired (n=71) | P value |
| Incubation period, M (IQR) | 2.00 (1.0,2.0) | 1.00 (1.0,2.0) | 0.177 | 2.0 (1.8,3.0) | 2.0 (1.0,3.8) | 0.335 |
| Disease recovery time, M (IQR) | 7.0 (5.0,14.0) | 7.0 (5.0,10.0) | 0.648 | 7.0 (5.0,15.0) | 5.0 (5.0,7.0) | ＜0.001 |
| Medical institution treatment, n (%) | 59 (78.7) | 25 (65.8) | 0.139 | 43 (60.6) | 32 (45.1) | 0.064 |
| Medication | 57 (76.0) | 15 (39.5) | ＜0.001 | 52 (73.2) | 53 (74.6) | 0.848 |
| Antiviral drugs | 35 (46.7) | 12 (31.6) | 0.124 | 22 (31.0) | 17 (23.9) | 0.347 |
| Antibiotics | 24 (32.0) | 6 (15.8) | 0.065 | 28 (39.4) | 30 (42.3) | 0.733 |
| Symptomatic treatment | 27 (36.0) | 9 (23.7) | 0.184 | 39 (54.9) | 51 (71.8) | 0.037 |
| Impact on work, n (%) |  |  | 0.077 |  |  | ＜0.001 |
| No influence | 1 (1.3) | 6 (15.8) |  | 4 (5.6) | 16 (22.5) |  |
| Less influence | 36 (48.0) | 17 (44.7) |  | 35 (49.3) | 39 (54.9) |  |
| Absence | 38 (50.7) | 15 (39.5) |  | 32 (45.1) | 16 (22.5) |  |

Figure legends：M, median; IQR, inter-quartile range.
